# Slow wave synchronization and sleep state transitions

**DOI:** 10.1038/s41598-022-11513-0

**Published:** 2022-05-06

**Authors:** Dan Guo, Robert J. Thomas, Yanhui Liu, Steven A. Shea, Jun Lu, Chung-Kang Peng

**Affiliations:** 1 Center for Dynamical Biomarkers, MA 02067 Sharon, USA; 2 Division of Pulmonary, Critical Care & Sleep, Department of Medicine, Beth Israel Deaconess Medical Center, Harvard Medical School, Boston, MA 02215 USA; 3 Olera Technologies, Inc., CA 94022 Los Altos, USA; 4 Oregon Institute of Occupational Health Sciences, Oregon Health & Science University, Portland, OR 97239 USA; 5 Department of Neurology, Beth Israel Deaconess Medical Center, Harvard Medical School, Boston, MA 02215 USA

**Keywords:** Neurophysiology, Mathematics and computing, Biological physics

## Abstract

Spontaneous synchronization over large networks is ubiquitous in nature, ranging from inanimate to biological systems. In the human brain, neuronal synchronization and de-synchronization occur during sleep, with the greatest degree of neuronal synchronization during slow wave sleep (SWS). The current sleep classification schema is based on electroencephalography and provides common criteria for clinicians and researchers to describe stages of non-rapid eye movement (NREM) sleep as well as rapid eye movement (REM) sleep. These sleep stage classifications have been based on convenient heuristic criteria, with little consideration of the accompanying normal physiological changes across those same sleep stages. To begin to resolve those inconsistencies, first focusing only on NREM sleep, we propose a simple cluster synchronization model to explain the emergence of SWS in healthy people without sleep disorders. We apply the empirical mode decomposition (EMD) analysis to quantify slow wave activity in electroencephalograms, and provide quantitative evidence to support our model. Based on this synchronization model, NREM sleep can be classified as SWS and non-SWS, such that NREM sleep can be considered as an intrinsically bistable process. Finally, we develop an automated algorithm for SWS classification. We show that this new approach can unify brain wave dynamics and their corresponding physiologic changes.

## Introduction

Synchronization of complex networks of interactive components is ubiquitous in nature, such as coupling of pendulums, flickering of fireflies, and social behavior of mammals^1^.

During non-rapid eye movement (NREM) sleep, synchronization of tens of billions of cortical neurons in the brain is a fundamental and crucial process, especially important for the initiation, maintenance and termination of deep sleep^2^. Deep sleep is also called slow wave sleep (SWS) because of the characteristic 0.3–4 Hz oscillations in human and animal electroencephalogram (EEG) that emerge from the faster EEG frequencies that define lighter stages of sleep. From the perspective of neuronal activity, after sleep onset, as slow wave synchronization is initiated, increases, and the amount of involved neurons exceeds a certain threshold, sleep state transitions from the light sleep to SWS occurs. Such a state transition may also be meaningful for vital physiologic activities such as respiratory and cardiac function, which are generally in their most stable (least variable) state during SWS. Indeed, we and others have previously observed a concordant alternating process appears at the transition in and out of SWS: as slow wave activity (SWA) increases above certain level, heart rate, respiration, and other physiologic variables become relatively stable^3,4^. As SWA gradually rises to its highest level, physiologic fluctuations remain at their minimal levels. After a period of time, the SWA deteriorates, and those previously stable physiologic variables exhibit a relatively abrupt switch to significantly larger fluctuations, as deep sleep switches to light or rapid eye movement (REM) sleep. We observed that such a sleep state transition involves multiple vital activities of the human body, from the central to the peripheral nervous systems. Based on our database, we hypothesized that such a transition pattern is universal, i.e., independent of age, gender, race, and conserved across species.

### Conventional criteria for sleep states

The most commonly used current sleep stage classification is the 2007 American Academy of Sleep Medicine (AASM) update^5^ of the 1968 Rechtschaffen and Kales (R&K) sleep categorization^6,7^. With these criteria, sleep staging is performed using visual and manual annotation^6^. REM sleep constitutes about 20% of sleep, and NREM sleep for the remaining 80%. The AASM rules further divide NREM sleep into Stages N1, N2, and N3, based on EEG morphology^5^. N3 sleep is considered as SWS, a crucial component of the sleep architecture. However, the scoring criteria are somewhat arbitrary (> 20% EEG SWA with amplitude ≥ 75 µV in a 30-s window^5,7^). For decades, various limitations of the manual sleep stage classification systems have been discussed: include the empirical threshold of SWA amplitude^8^, low temporal resolution^8,9^, weak correlation between electrophysiological activity and stages, ignorance of other physiological parameters, etc.^9^ Therefore, other sleep classification approaches have been proposed. For example, cyclic alternating pattern (CAP) and non-CAP sleep have been studied extensively^10,11^, and depicts NREM sleep as a two-state process based on visual EEG scoring. Similarly, an electrocardiogram-based cardiopulmonary sleep analysis also reported a bistable state transition during sleep^12^.

### Puzzles emerging from the use of conventional sleep stages

According to current scoring criteria, in the elderly population, N2 sleep is the most common NREM stage, and there is little or no deep N3 sleep, partly because the slow waves in the elderly do not meet amplitude criteria^13^. However, during the sleep that follows a period of sleep loss, there is a clear SWA ‘rebound’ in both young and elderly humans^14^, with little difference between these groups in the SWA rebound dynamics (e.g., rebound proportion and decay slope), albeit from lower absolute SWA amplitude in the elderly compared to the young population^15^. In addition, loss of sleep in later-part of the night in elderly humans will similarly induce a SWA rebound during subsequent sleep, indicating that SWS debt is being built up even though this period is usually dominated by N2 sleep^15^.

Conventional sleep stages do not take into consideration the physiological hysteresis that accompanies N2 sleep transition. For instance, N2 sleep that precedes REM sleep is associated with increasing stress-related biomarkers, while N2 sleep that precedes N3 sleep is associated with significantly decreasing stress-related biomarkers^16^.

From a clinical perspective, while many diseases affect sleep, there are usually very weak correlations between the severity of the disorder and the amount of N3 sleep^17,18^. These observations suggest that, rather than the current N3 sleep classification, other characteristics of SWA may be more physiologically or clinically relevant.

Therefore, here we present a different framework first focusing only on NREM sleep. We view SWS as a synchronization process, and thus divide NREM sleep into slow wave non-synchronized and synchronized states. Applying this fundamental concept, we develop a fully automated computational algorithm that can re-classify NREM sleep into SWS and non-SWS states in healthy people without sleep disorders. We show that this new classification framework may begin to resolve aforementioned limitations or contradictions.

### Empirical mode decomposition (EMD) analysis of sleep EEG

**Figure 1:**
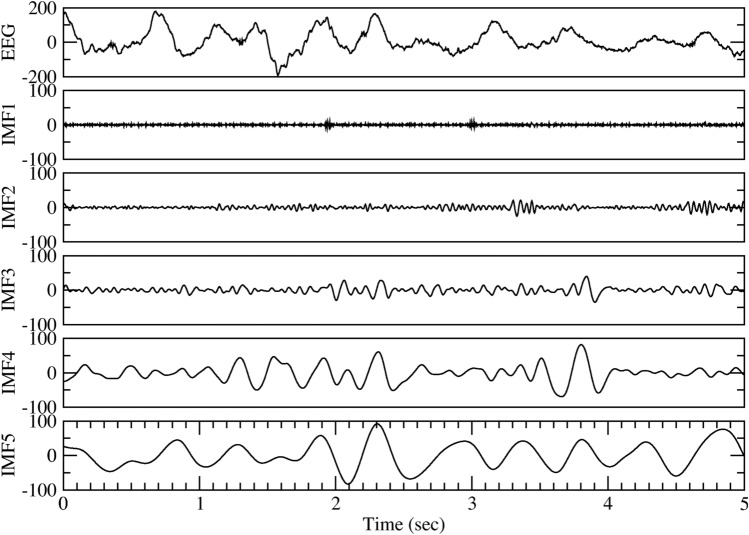
EEG slow wave activity can be separated as independent time series by empirical mode decomposition (EMD). Five seconds of single channel EEG signal during slow wave sleep (SWS) is shown in the top panel. The bottom 5 panels show the first 5 intrinsic mode functions (IMF1 to IMF5) extracted by EMD. The frequency range for IMF3 to IMF5 is within the slow wave oscillations frequency, thus a large amplitude of these 3 IMFs indicates the presence of SWS.

To detect synchronization patterns in the EEG signals, we need an accurate analysis tool that can separate the SWA from other fluctuations. To this end, we applied the empirical mode decomposition (EMD) analysis, which can decompose complex signals into a collection of intrinsic mode functions (IMF)^19,20^. Each IMF is an oscillatory function with a time-varying amplitude and frequency. The advantage of EMD is that it is applicable to nonlinear and nonstationary signals without assuming any a priori basis function^19–22^. Detailed description about the EMD method can be found in the Supplementary Material. Figure 1 shows a 5-s EEG segment, and the first five IMFs by the EMD decomposition. The first IMF (denoted as IMF1) of EEG has the highest frequency, the last IMF has the lowest frequency, and slow waves can be found distributed in some of the modes. EEG signals used in this study are sampled at 120–125 Hz, as a result, IMF3, 4, 5 contain oscillations in frequency range 0.2–4 Hz (see Supplementary Material for detailed explanation), i.e., the frequency band of slow waves (Fig. 1). We can thus combine IMF3, 4, and 5 and define the total SWA by calculating the amplitude of the combined signal.

### Bistability of NREM sleep

During an overnight sleep, the brain usually achieves a synchronized SWS state for some periods of the night. We hypothesize that this state transition can be modeled by a stochastic process of forming a synchronized neural cluster. Such a stochastic process of an emerging cluster of neural synchronization can be represented by a simple stochastic model, with the cluster size at time $\mathrm{t}$, denoted as $\mathrm{S}(\mathrm{t})$, to fluctuate in time, with the following rule:

$$\mathrm{S}\left(\mathrm{t}+1\right)=\mathrm{S}\left(\mathrm{t}\right)+\Delta \mathrm{S},$$

where $\Delta \mathrm{S}$ is a random variable. This naive model simply states that the cluster size can increase (if $\Delta \mathrm{S}>0$), decrease ($\Delta \mathrm{S}<0$), or remain the same ($\Delta \mathrm{S}=0$) at each time step stochastically. If we consider the case that the random variable $\Delta \mathrm{S}$ is independent of the cluster size $\mathrm{S}(\mathrm{t})$, e.g., $\Delta \mathrm{S}$ is centered around zero with an arbitrary pre-defined distribution, then this is the standard “random walk” model^23^. The cluster size distribution will approach Gaussian (normal) distribution, as governed by the central limit theorem^23^. However, a more realistic model should consider the random variable $\Delta \mathrm{S}$ proportional to the cluster size $\mathrm{S}(\mathrm{t})$, e.g., $\Delta \mathrm{S}\propto \mathrm{S}(\mathrm{t})$, therefore,

$$\mathrm{S}\left(\mathrm{t}+1\right)=\mathrm{S}\left(\mathrm{t}\right)+\updelta \cdot \mathrm{S}\left(\mathrm{t}\right)=\mathrm{S}\left(\mathrm{t}\right)\cdot \left(1+\updelta \right),$$

where $\updelta$ is a random variable centered around zero. Taking the logarithm of the above equation, we obtain

$$\mathrm{logS}\left(\mathrm{t}+1\right)=\mathrm{logS}(\mathrm{t})+\mathrm{log}\left(1+\updelta \right),$$

we can treat $\mathrm{log}(1+\updelta )$ as a new random variable, and thus the stochastic process generating $\mathrm{logS}(\mathrm{t})$ (i.e., the logarithm of cluster size) is also corresponding to a random walk model. It implies that the neural cluster grows or decays exponentially at each instant, such that the cluster size would be normally distributed on the logarithmic scale, i.e., log-normal distribution, when sampled over time.

This simple synchronization model implies that if we examine the histogram of SWA amplitude, an indirect measure of the synchronized cluster size, then we should observe the hint of two log-normal distributions, one of them is centered at a large SWA amplitude, corresponds to the fully synchronized state, while the other (with smaller SWA amplitude) corresponds to the non-synchronized state.

**Figure 2:**
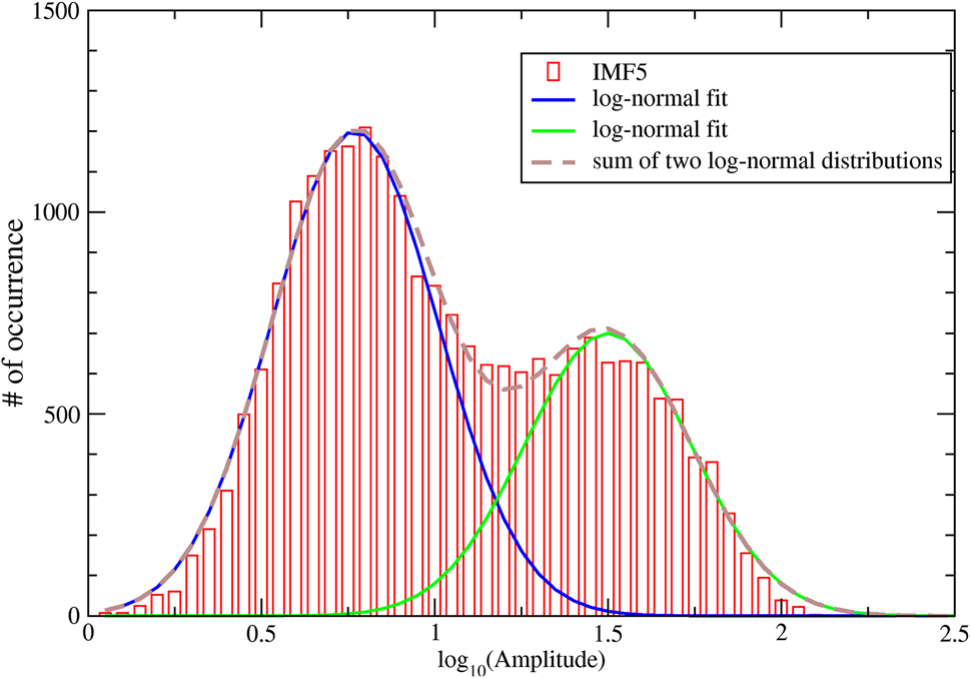
Histogram of IMF5 amplitude for an entire night NREM sleep of a healthy 25-year-old female subject. The average amplitude of IMF5, i.e., the main component of SWA, for every 2-s segments is calculated. The histogram is plotted on a semi-log graph, and exhibits a bimodal distribution that can be nicely fitted by the superposition (dashed brown curve) of two log-normal distributions (solid blue curve and dashed green curve).

Figure 2 shows the histogram of SWA amplitude (measured for every 2-s period) for NREM sleep over an entire night in a typical healthy young subject. A bimodal distribution was observed as predicted by our simple synchronization model. The graph supports two important outcomes of our model. First, it indicates that there are two different states, in terms of SWA amplitude: one with high SWA amplitude (denoted as the SWS state), and one with low SWA amplitude (denoted as non-SWS state). Second, the histogram can be nicely fitted by the superposition of two log-normal distributions, which confirms that the underlying process of synchronization can be described by our simple model.

**Figure 3:**
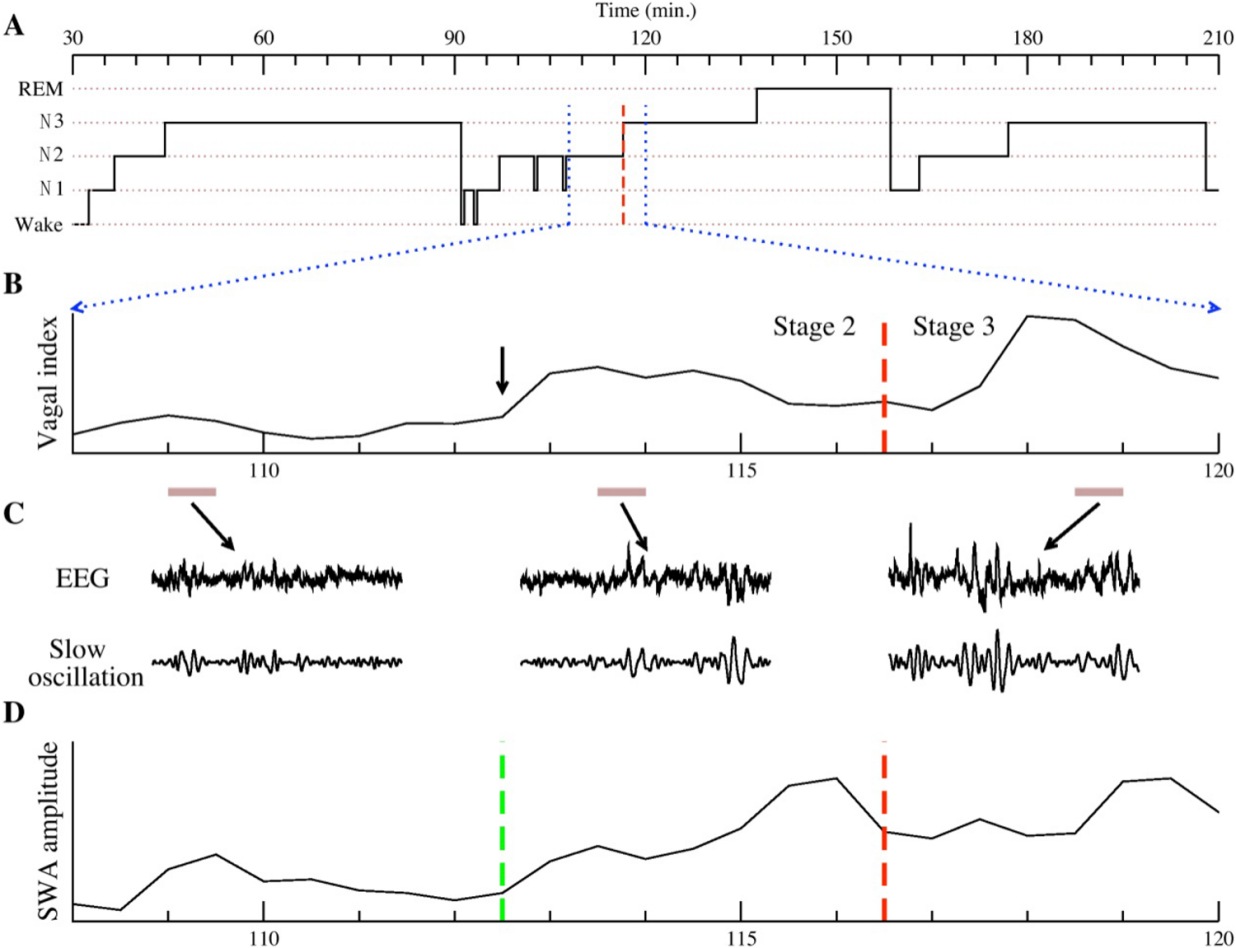
(**A**) Conventional AASM sleep stage annotation for a 3-h sleep period. We focus on the 12-min sub-period enclosed by the two vertical dotted blue lines (from 108 to 120 min) that contains both stages N2 and N3. A dashed red vertical line indicates the transition from N2 to N3. (**B**) A heart rate derived cardiac vagal tone indicator, as measured by the ratio of high- to low-frequency heart rate variability, is plotted for this period. Of note, cardiac vagal tone starts to rise (arrow) earlier than the annotated transition to N3 (red dashed line). (**C**) A “blow-up” of the EEG of three 30-s periods, indicated by brown horizontal bars, are presented. These include two periods from N2 (one with basal vagal tone, the other with elevated vagal tone), and one period from N3. To better visualize SWA in the EEG, we plot, underneath the raw EEG, one intrinsic mode function (IMF) that corresponds to the oscillatory component in the 0.3–1 Hz band. This plot shows that SWA appears, albeit with lower amplitude, prior to the N3 transition. (**D**) An EMD-based SWA amplitude is computed and plotted for the 12-min period. An algorithm was developed to identify the “transition point” (green vertical dashed line) when SWA starts to emerge. This SWA transition point appeared much earlier than the N2 to N3 transition (red dashed line). As we will show, this transition point that is associated with cardiac vagal tone transition (SWA usually rises about 2 min earlier than the elevation of vagal tone), and suggests that the EMD analysis of SWA provides a physiologically relevant definition of slow wave sleep.

**Figure 4:**
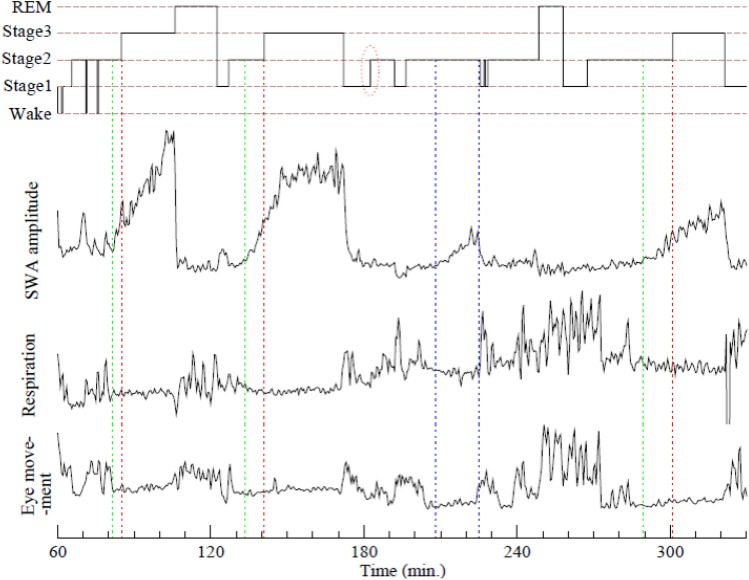
A typical sleep stage annotation of a 4.5-h sleep period is shown in the top panel. In the other three panels, amplitude of SWA, respiration (amplitude of breathing flow), and eye movements (amplitude of EOG signal) during the same period are plotted. Three dotted red lines mark transitions from N2 to N3 according to AASM criteria. SWA amplitude consistently increased prior to these transitions and our algorithm identifies the earlier transition points (green lines) when SWA emerges and becomes dominant. We hypothesize that these transition points identify when synchronized networks of neurons are formed, and might represent a more meaningful transition to slow wave sleep (SWS) as this is concordant with other physiologic variables, as evidenced by the lower two panels. The two blue lines identify an SWS period inside a conventional N2 stage.

Such a bimodal description of NREM sleep, based on SWA amplitude, is quite different from the 4 or 3 NREM stages based on older or current heuristic classification systems (R&K and AASM rules, respectively). However, there are interesting patterns between the synchronized SWS state and the conventional classification schemes (see Fig. 3). Using the AASM rules as an example, SWA usually starts during N2 sleep and reaches its highest level in N3 sleep, then disappears abruptly when N3 is terminated. Some relatively low peaks of SWA also appear in N2 without reaching the 20% criteria for classification as N3 stage (the zone defined by the two blue dotted lines in Fig. 4). This observation leads us to develop an automated algorithm that can classify these two states, i.e., SWS vs. non-SWS, without human intervention. See the “Materials and methods” section for technical details of the computational algorithm.

### Unify brain wave dynamics with other physiologic changes

Compared to conventional scoring of sleep stages, the new algorithm provides a more consistent picture for sleep state transitions and is aligned with the dynamics of other physiologic variables. Specifically, the synchronized SWS state is accompanied by a rise of vagal tone, and with stable heart rate, breathing flow, as well as electromyogram (EMG) and electrooculogram (EOG), etc. (see Fig. 4 for some of the signals). When the synchronization is disrupted, vagal tone plunges and all other quiet signals suddenly exhibit large fluctuations simultaneously. We observed such dynamics of SWA and the concordance with other physiologic signals in all subjects, including elderly subjects with little or no N3.

### The duality of N2 sleep resolved

Applying our SWS classification algorithm, N1 is classified as non-SWS, N3 is classified as SWS, while N2 is divided into two sub-groups, corresponding to non-SWS (denoted as N2a) and SWS (denoted as N2b), respectively. When SWA emerges—with amplitude higher than the background noise—in N2b, although the intensity is much lower than the conventional criteria of 75 µV, the vagal tone has increased into a level similar to that in N3 sleep. Some N2 sleep periods prior to N3 sleep, such as the N2b period in Fig. 3, has already exhibited SWS characteristics. Moreover, cardiac vagal tone in N2b sleep is significantly higher than during N2a sleep (see Fig. 3, Fig. S1).

Thus, to seek reliable coincident physiological indicators for the separation of N2a and N2b sleep, we generated a vagal tone index every 30 s of full-night data in 73 subjects with artifact-free ECG signals. The average vagal intensity of N2b sleep is significantly higher than that in N2a, N1 and REM sleep (p < 0.01), but similar to that of N3 sleep. Furthermore, no statistical difference was found (p > 0.1) for cardiac vagal tone between N2a sleep and N1 sleep. We also examined this relationship with Bayesian statistics and found Bayes factor is 0.097, indicating that the observed data are 10.35 times more likely under the null hypothesis than under the alternative hypothesis. This is strong evidence for the null hypothesis that there is no difference on the vagal intensity between N2a sleep and N1 sleep. These observations for cardiac vagal tone are consistent to the classification by our new algorithm using SWA, i.e., N2a is non-SWS as N1 sleep, but N2b is SWS as N3 sleep. Thus, the previously unexplained duality of N2 sleep appears better resolved with consideration of the concordance with cardiac vagal tone.

### Quantify SWS in different age groups

**Figure 5:**
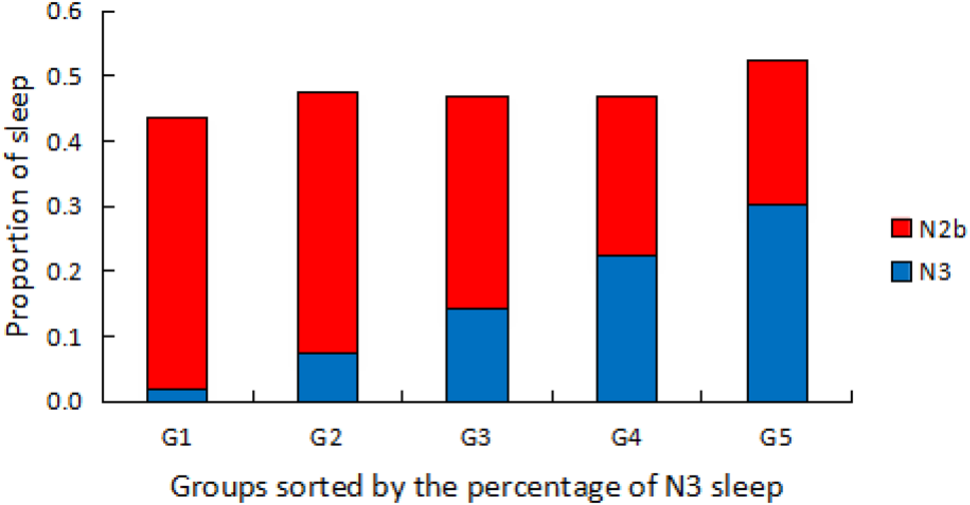
Forty six healthy elderly subjects were divided into 5 quintiles according to percentage of N3 sleep. Group 1 has the lowest amount of N3 sleep, and group 5 has the highest. The average percentages of N3 sleep for each group are plotted in blue. The ‘hidden’ slow wave sleep (SWS) periods in N2 sleep, denoted as N2b, uncovered by our algorithm are plotted in red. Note, as the amount of N3 sleep increases from group 1 to 5, the amount of N2b decreases, leading to an almost constant percentage of the sum of N3 and N2b of 45–50% across all 5 subgroups. The proportions of SWS in NREM sleep of these 5 subgroups are not statistically different (p > 0.9, One-way ANOVA).

With our new approach, in 46 healthy elderly subjects with 15.3% conventionally scored N3 sleep, an additional 32.2% SWS using our approach is “uncovered” from the AASM scored N2 sleep. Thus, the actual total SWS is about 47.5% for this group. We divided the 46 subjects into 5 quintiles based on the percentage of N3 sleep, the average N3 sleep are 2.0%, 7.6%, 14.1%, 22.4%, 30.4% and the “uncovered” N2b sleep was 41.5%, 39.9%, 32.9%, 24.6% and 22.1%, respectively. The actual total SWS of the 5 groups were 43.5%, 47.5%, 47.0%, 47.0% and 52.5%. Thus, the total SWS maintained at a stable narrow range across these 5 sub-groups (Fig. 5). The proportions of traditional AASM scored N1, N2, N3 sleep are plotted in Fig. S2.

Similar results were found for a healthy young group with 59 subjects (Supplementary Materials Figs. S3, S4). However, the average amount of total SWS assessed by our technique was actually lower (38%) than in the elderly (47.5%). We also found that N1 sleep of the young group was much higher (16.0%) than in the elderly (5.5%) These results reinforce our finding that total SWS does not appear to decline with age when assessed by our technique. We do note that for the elderly group, all sleep studies were completed at home; whereas for the young group, all sleep studies were performed in a sleep laboratory. Thus, these unexpected differences between groups in the proportions of sleep stages could have been partly or entirely caused by the ‘first-night effect’ whereby sleep is slightly more disturbed when people are exposed to an unfamiliar environment, as occurred in the younger group^24^. The mechanism has been speculated to imply increased vigilance in one hemisphere associated with the first-night effect in human sleep^25,26^.

## Discussion

Based on the visual scoring of EEG waves, conventional AASM sleep criteria categorizes NREM sleep into N1, N2 and N3 sleep stages. This classification scheme encounters three key ambiguities: (1) N2 stage is inconsistent with other major physiologic responses^12,16^; (2) there is a duality of N2 sleep^16^; (3) There is a dramatic decrease of N3 sleep in healthy elderly^13,14^. N3 sleep, or in general, SWS has been studied extensively in the past few decades. Slow wave activities are thought to be involved in endocrine regulation^27^, memory consolidation^28–31^, synaptic homeostasis^32–34^, and the restorative function of sleep^35^. Based on the analysis of slow wave activities, our framework of classifying NREM sleep into synchronized (SWS) vs. non-synchronized (non-SWS) states resolves the above three ambiguities. Specifically, with our algorithm: (1) a more consistent physiologic association is demonstrated between sleep state transitions and other physiological changes; (2) N2 sleep periods are divided into SWS and non-SWS which addresses the “duality of N2 sleep”; and (3) the total amount of SWS is relatively stable in all healthy adults, both for the young and the elderly groups. This last observation differs from the conventional view that there is “little SWS in the elderly group”^13^. Indeed, our observation that SWS percentages are similar in the healthy young and elderly groups indicates that the amount of SWS appears to be well preserved across our lifespan, just like REM sleep constitutes consistently about 20% of sleep. However, the micro-structure of SWS certainly does change with age. For example, the SWA intensity and the transition dynamics of SWS in the elderly have different characteristics from that in the young^36,37^. We also observed visually that, in agreement with findings by previous research studies^36,37^, SWA intensity decreases and the fragmentation of SWS increases in the elderly group, with a less-steep slope of the up and down phase.

Our study provides a novel conceptual framework of modeling SWS as a synchronization process of a network of coupled oscillators. During an overnight sleep, the brain tries to reach the synchronized SWS state, and we hypothesized that it can be modeled by a stochastic process of forming a synchronized neural cluster. Our model also predicts that the cluster size would follow log-normal distribution, as confirmed by some of the data.

A first-order simplification of the model that focuses only on the amplitude of SWA allows us to divide sleep into SWS state and non-SWS state. The SWA recorded from surface EEG reflects the synchronization of the extracellular electrical activity in the deeply “resting” cortex. With arousal system deactivated and the sleep system activated, sleep initiates. When more neurons join a synchronized network, stronger SWA appear on surface EEG, reflecting a relatively stable state with high intensity synchronization. Based on our observation of the dynamics of SWA, the following process may take place: When the amount of hyperpolarized neurons reaches a certain threshold, 0.2–4 Hz synchronized activity becomes the dominant oscillation in our brain, SWS emerges; from this point on, more and more neurons are recruited into this oscillating storm, thus boosting SWA to reach a relatively stable state with high intensity. Eventually, with the sleep promoting neurons being inhibited to some extent and arousal factors increasing, the synchronization collapses and disappears quickly, such that SWS sleep deteriorates into non-SWS sleep, REM sleep or wake. The above picture may also account for the asymmetric property of SWA, namely that SWA gradually increases in the beginning of SWS, but terminates abruptly from a relatively high intensity.

Since the forming of a synchronization cluster is a stochastic process, it could be affected by many uncontrollable factors: the amount of time a dominant cluster is formed, the size of the maximal synchronized cluster, noisy perturbations from the environment, etc. Therefore, it is unlikely that all data will display a clear bimodal distribution that two log-normal distributions are easily visible. For example, if the SWA amplitude of the SWS state is not much greater than that of the non-SWS state, then the two distributions may merge into one. Therefore, without a clear sign of bimodal distribution does not mean that the two states (SWS vs. non-SWS) are not there, as it is reasonable to believe that the underlying synchronization process remains valid. This theoretical consideration motivates us to develop our automated algorithm to identify SWS periods. See Supplementary Materials for detailed discussion on how our algorithm can uncover the two underlying “hidden” distributions for some data.

As a proof of concept, we also found that it is possible to apply our algorithm—without major modification—to classify SWS in rats. Moreover, consistent with the results in humans, analysis of EEG in rats also revealed a bimodal distribution of SWA (Fig. S6), suggesting that NREM sleep in rats also consists of two easily definable states. Thus, we speculate that a bimodality of NREM sleep involving a synchronization process of a network of coupled oscillators could be a more universal phenomenon that is evolutionarily conserved.

Sleep-related EEG oscillations are relatively slow, including slow oscillation (0.3–1 Hz), delta (1–4 Hz), spindle (7–14 Hz), etc.^38^, which together initiate and maintain sleep, involved in memory consolidation and neural plasticity during sleep^31,39,40^. Our new classification algorithm focuses almost exclusively on the difference between SWS and non-SWS, and it does not address the entire complex architecture of sleep, such as sleep spindles, K-complexes, other fast rhythms beyond slow waves^41,42^. Nonetheless, in addition to SWA, two other frequency components were also considered in our classification algorithm: rhythms faster than 20 Hz (fast oscillations), and rhythms slower than 0.1 Hz (infra-slow oscillations). Oscillations faster than 20 Hz are associated with an activated state, such as wake and REM sleep. Fast rhythms appear transiently in the depolarizing phase of sleep as well, but are enhanced within intracortical networks during brain activation^43,44^. We found that fast components increase not only in wake and REM sleep, but also during unstable NREM sleep (non-SWS). However, these faster components stay at a relatively low and steady level only during stable SWS (Fig. S5). This observation is consistent with the hypothesis that fast rhythm intensity serves as an indicator for degree of consciousness.

As for the infra-slow oscillations in the EEG, which were oscillations within 0.01–0.1 Hz in our algorithm, these oscillations are essentially ignored in conventional sleep stage characterization as the AASM rules recommend 0.3 Hz as the high-pass threshold of EEG^5^. Thus, oscillations slower than 0.3 Hz are usually filtered out and not fully investigated^45^. When we examined the unfiltered EEG (full-band EEG), we found that infra-slow oscillations robustly fluctuate in the opposite direction of SWA (Fig. S5) in all subjects, which is consistent with a previous finding^46^. Such relatively long time scale oscillations match well with the average duration of CAP (20–40 s) in NREM sleep^10,11^. CAP has been recognized as the marker of sleep instability and may be enhanced by the internal or external interference^10^. Further investigations are needed to verify the connection between infra-slow oscillations and CAP.

In this regard, the normalized ratio (see definition in the “Materials and methods” section) between SWA and infra-slow oscillations plus fast oscillations reflects the balance between sleep enhancing and arousal enhancing processes. When the normalized ratio is higher than 1, SWS occurs, whereas when the ratio is lower than 1, sleep consolidation is impaired due to the interference from arousal factors, yielding a state of unstable sleep (or non-SWS).

For over six decades, sleep staging has mostly relied on manual review and annotation of paper or computer screen polysomnograms. A night of sleep requires at least an hour of scoring by trained personnel. However, epoch (30 s) by epoch agreement between scorers is typically less than 80%^47^. The most common discrepancies using AASM criteria occur between classifications of N2 and N3 sleep (followed by the discrepancies between N1 and N2 sleep)^48^. Such inter-scorer differences are likely due to both human bias and the inherent arbitrariness of the scoring criteria. In contrast, our automated SWS classification algorithm is entirely automatic without arbitrary thresholds. Furthermore, the analysis does not rely on expert judgement, and thus avoids inter-scorer discrepancy. Nonetheless, although we compared our new algorithm to the conventional AASM criteria, it is not our intention to suggest our approach as a replacement of the current AASM standard. As noted above, our classification algorithm does not address the entire complex architecture of sleep, including REM sleep, N1 sleep and N2a components of non-SWS. Furthermore, since all current database are healthy subjects, the new algorithm has not been applied to subjects with sleep disorders that may lead to very little or absent of SWS. For those conditions, further refinement of our algorithm is needed.

In summary, our goal is to introduce a critical examination of SWS that has not been fully considered and meaningfully quantified, and in concert to look for other physiological correlates of the new quantification. NREM sleep may be best conceptualized and quantified as a bistable process based on the synchronization and desynchronization of slow oscillations. The detailed neurophysiologic mechanism of this transition remains to be determined, but raises the intriguing possibility of as yet poorly defined neural dynamics that regulate both cortico-thalamic as well as autonomic circuitry. We believe this initial step of proposing a different perspective on sleep structure will help us move towards a more comprehensive understanding of sleep. For a full understanding, this SWS/non-SWS oscillator should also be considered in conjunction with a REM/non-REM oscillator and the longer time scale sleep–wake oscillator.

## Materials and methods

### Databases

105 subjects with conventional sleep stage scoring, covering a wide age range as described below.

1. 59 healthy young to middle age subjects (20 females and 39 males), average age 26.9 ± 7.8, with the following age distribution. 18–30 years old: 44 subjects; 31–40 years old: 11 subjects; 41–50 years old: 4 subjects. 31 of these young subjects were healthy volunteers who had responded to a recruitment to participate in studies of circadian physiology in the Division of Sleep Medicine, Brigham & Women’s Hospital in 2004^49^. Their health status was confirmed by extensive medical history questionnaires followed by electrocardiography, blood chemistry profiles, liver function tests, complete blood count, urinalysis, a history and physical examination by a physician, and a psychiatric and psychological examination by a clinical psychologist. Subjects were excluded if they were obese (body mass index [BMI] > 30); taking any medications; or had any chronic or current acute medical or psychiatric disease. These subjects underwent full nocturnal polysomnography monitoring, and sleep stage scoring was performed by registered technicians at laboratories accredited by the AASM. The other 28 of the young subjects were healthy volunteers who had responded to an advertisement to participate in a project of sleep physiology in Beijing Chinese Medicine University in 2016 in accordance with institutional guidelines. Their health status was established by medical history and an overnight polysomnography recording. The exclusion criteria was the same as the above. A single overnight polysomnogram using the Alice PDx system was performed in regular hotels inside or near the University. The sleep stage scoring was performed by registered technician accredited by the AASM.
2. 46 healthy elderly subjects (32 females and 14 males), average age 67.6 ± 5.6 years old, with the following age distribution. 60–70 years old: 34 subjects; 70–80 years old: 12 subjects. These subjects were selected from the baseline study of Sleep Heart Health Study (SHHS) database^50^. Their health status was established by medical history and life quality questionnaires. In SHHS, Compumedics PS polysomnograph was applied to complete a single over-night polysomnogram. The montage includes oximetry, electrocardiogram (ECG), chest wall and abdominal movement, nasal/oral airflow by thermistor, body position, EEG, electrooculogram (EOG), and chin electromyogram (EMG). Subjects were excluded if they had the following medical conditions: ischemic heart disease, heart failure, asthma, chronic obstructive lung disease, hypertension, diabetes and stroke, or the polysomnogram showed an apnea hypopnea index ≥ 5/h of sleep or respiratory disturbance index (events scored without desaturation) ≥ 15/h of sleep. Using these criteria, 380 healthy subjects was selected. Subjects using any sedatives or hypnotics, use of alcohol, more than 5 cups of coffee or tea a day were also excluded. In total, 46 subjects were selected as the relative healthy elderly group.

### EEG metrics

1. Empirical mode decomposition (EMD) is an adaptive decomposition method for separating different modes of frequency and amplitude modulations in the time domain^21,22^. We applied EMD to decompose the EEG signal into a finite number of intrinsic mode functions (IMFs). Each IMF represents a narrow-band amplitude and frequency-modulated signal. In our study, the sampling frequency of the raw EEG signal is ~ 120 Hz, we found the frequency range for the decomposed IMF3 is about 2–4 Hz, and 1–2 Hz for IMF4, 0.2–1 Hz for IMF5. Thus the combined amplitude of IMF3, 4, 5 (0.2–4 Hz) is an accurate measure of the intensity of SWA. Also, the frequency range for IMF1 is about 20–60 Hz and the amplitude of IMF1 represents the intensity of fast oscillation. The frequency range for IMF8, 9, 10 are about 0.01–0.1 Hz and the combined amplitude of IMF8, 9, 10 (0.01–0.1 Hz) represents the intensity of infra-slow oscillation.
2. Distribution of SWA. We use a window of 2 s long to calculate the average amplitude of SWA, then we move the window by 1 s to calculate the average amplitude again. The average amplitudes for all 2-s windows of the entire NREM sleep were used to get the SWA amplitude distribution. We inspect the SWA amplitude distribution of all subjects we studied to explore whether they are bimodal distributions.
3. SWS classification. We noticed that when a subject is in SWS (with all corresponding physiologic signals stable), the SWA amplitude increases as expected, but additionally, the amplitudes of IMF1 (fast brain wave) and IMF8, 9, 10 (may correspond to CAP related oscillation, an indication of unstable sleep) decrease. We conclude that the amplitude of combined IMFs 3, 4, and 5 can serve as an index for SWA strength; while in the opposite direction, the amplitude of the summation of IMFs 1, 8, 9, and 10 is a good index for non-SWA strength. We use a window of 60 s to calculate these two indices (SWA strength and non-SWA strength) for that window. Furthermore, we need to consider individual differences in EEG signals (for example, young subjects usually have larger amplitudes than elderly subjects), therefore, we measure the global median values of SWA and non-SWA for each subject over his/her entire NREM sleep. Then we divide these two local indices (calculated for a window of 60 s) by their global median values to normalize these two indices. Finally, a normalized ratio, i.e., normalized SWA strength divided by normalized non-SWA strength, is calculated. When the normalized ratio is greater than or equal to 1, SWA dominates the system, so we classify it as SWS state; otherwise, non-SWS state. The window is then move forward by 30 s to determine the sleep state for the next time period. We use 30-s sliding step so that we can match the epoch length in conventional sleep stage scoring. In addition, to test the robustness of the threshold, we increased the threshold by 10% (i.e., set the threshold to be 1.1), as well as decreased the threshold by 10% (i.e., set threshold to be 0.9), and observed similar qualitative results.
4. We applied our classification algorithm to all 105 subjects. SWS quantified in both conventional N2 and N3 sleep constituted the total SWS amount.
5. Characteristics of SWS were compared with conventional N3 sleep. Subjects were sorted according to the N3 sleep percentage and evenly grouped into 5 sub-groups from low to high. Average SWS was described and compared with N3 sleep in the 5 sub-groups, as a comparison between our SWS and conventional deep sleep.

### HRV analysis

1. Since some of the conventional N2 sleep periods have been divided into non-SWS and SWS segments, denoted as N2a and N2b, respectively, according to our new classification rule, we are interested to make pair-wise comparison of heart rate variability (HRV) indices, to see if N2a and N2b have different physiologic characteristics. To this end, we identify N2 periods that meet the following criteria: (a) both N2a and N2b segments exist in the same N2 period; (b) both N2a and N2b segments have to be longer than 4 min (to ensure reliable HRV measurements). A total of 85 N2a–N2b pairs were identified from a subset of subjects (including both young and elderly subjects). ECG in these selected periods exported from polysomnographic recording was analyzed by a beat detection program to generate intervals between consecutive ECG R waves (RRI). The conventional HRV indices were calculated, including standard deviation (SD), power in high frequency band (HF), low frequency band (LF), very low frequency band (VLF), HF/LF ratio, HF/(LF + VLF) ratio. The pNNx index was also computed, which is the percentage of normal heartbeat with RRI that is x milliseconds more than the neighbor RRI. pNNx is recommended as a more robust index to reflect vagal activity^51^.
2. In all 105 subjects, full-night ECG was exported from the polysomnogram and analyzed by a beat detection program to generate RRI. ECG quality (the ratio of RR period over the length of the record) less than 80% was excluded. In total, 73 subjects were further analyzed. Similar to that in part (1), the conventional HRV index HF/(LF + VLF) was generated with a 30-s window. The HRV difference among sleep stages was compared.

### Statistics

Paired t-test, ANOVA and Bayesian statistics were used to compare HRV indices among different sleep stages. One-way ANOVA was applied for the SWS percentages among multi-groups. Bayesian factors are generated for the SWA amplitude comparison. The software is IBM SPSS 28.0.0.0.

All methods were carried out in accordance with relevant guidelines and regulations and the experimental protocols of data acquisition were approved by institutional review board (IRB) of Brigham & Women’s Hospital^49^ and Beijing Chinese Medicine University (the young subjects group). For the elderly subjects from the database of SHHS, IRB approval was obtained by each investigative center locally^50^. Informed consent was obtained from all subjects.

## Supplementary Information

Supplementary Information.
