# Supplementary material for: Slow wave synchronization and sleep state transitions
Source: Sci Rep. 2022 May 6;12:7467. doi: 10.1038/s41598-022-11513-0 (PMC9076647; doi:10.1038/s41598-022-11513-0)
Supplement: Supplementary file 1 — Supplementary Information. [file 41598_2022_11513_MOESM1_ESM.docx]

**Supplementary Materials**

To find further evidence for the separation of N2a and N2b sleep, we carried out the following analysis. First, we identified N2 periods that meet the following criteria: (a) both N2a and N2b segments exist in the same N2 period; (b) both N2a and N2b segments are longer than 4 minutes (to ensure reliable heart rate variation [HRV] measurements). A total of 85 N2a-N2b pairs were identified from a subset of subjects (including both young and elderly subjects). The comparison of these 85 N2a-N2b pairs shows that all conventional heart rate variation (HRV) indices have significant difference between these two sleep states (see the Methods section for details of HRV indices). All HRV indices support the concept that cardiac vagal tone is significantly higher in N2b sleep than that in N2a sleep (Fig. S1). These observations are consistent to the classification by our new algorithm, i.e., N2a is non-SWS and similar to N1 sleep, but N2b is SWS and similar to N3 sleep.


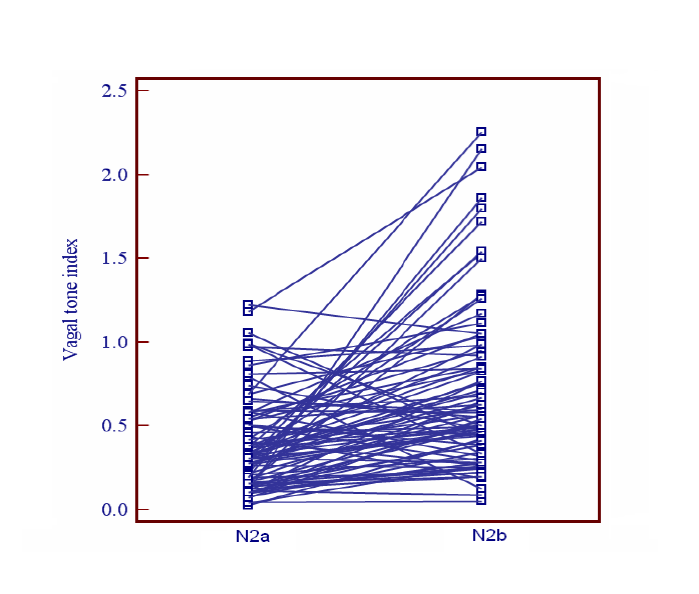


**Figure S1** Total of 85 N2a-N2b pairs were plotted here: the comparison of typical vagal tone index HF / LF ratio between N2a and N2b sleep. The vagal tone during N2b is significantly higher than that in N2a (p<0.001).


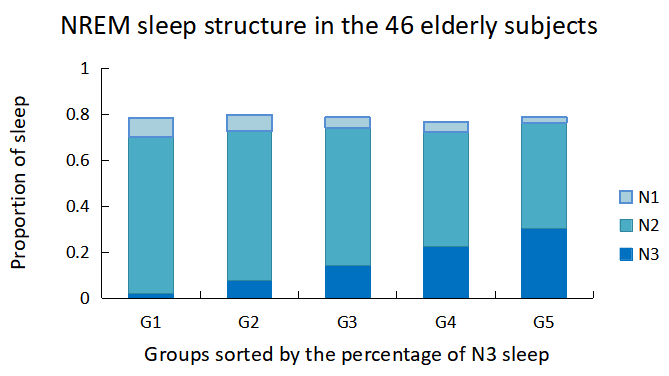


**Figure S2** For the 46 elderly subjects group, conventional sleep stages N1, N2 and N3 were plotted with ascending N3 sleep percentage. N1, N2 and N3 sleep together constitute a relatively stable NREM sleep percentage, 80% of total sleep time. Sleep studies were completed at home in this group. The average of N1, N2, N3 sleep for the group are 5.5%, 57.7% and 15.3%, respectively.


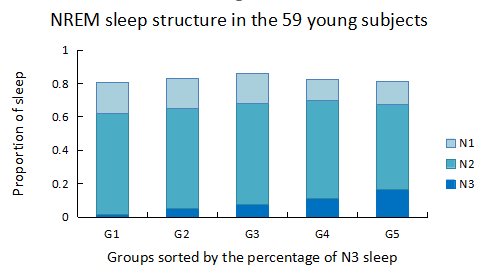


**Figure S3** For the 59 young subjects group, conventional sleep stages N1, N2 and N3 were plotted with ascending N3 sleep percentage. N1, N2 and N3 sleep together constitute a relatively stable NREM sleep percentage, 80% of total sleep time. Sleep studies were completed in a sleep lab or hotel in this group. The average of N1, N2, N3 sleep in this group are 16.1%, 58.3% and 8.3%, respectively. Light sleep N1 is much higher than that in the elderly group (5.5%), likely due to first night effect.


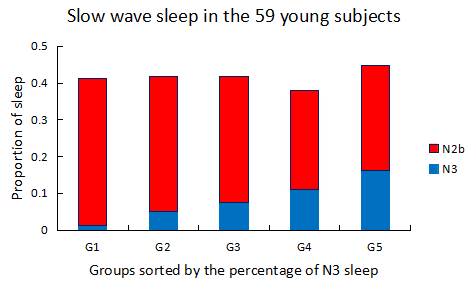


**Figure S4** Fifty nine healthy young subjects were divided into 5 quintiles according to percentage of N3 sleep. Group 1 has the lowest amount of N3 sleep, and group 5 has the highest. The average percentages of N3 sleep for each group are plotted with blue vertical bars. The hidden slow wave sleep (SWS) periods in N2 sleep, denoted as N2b, discovered by our new algorithm are plotted with red vertical bars. Note that as the amount of N3 sleep increases from group 1 to 5, the amount of N2b decreases accordingly, leading to a relatively constant percentage of the sum of N3 and N2b at around 38.0-42.0% across the 5 subgroups. The average amount of total SWS in the young was about 41.5%, lower than that in the elderly (47.5%, Fig. S2); N1 sleep of the young group was 16.1%, much higher than that in the elderly (5.5%). Such a difference could be the result of the first night effect since the young subjects completed their sleep studies in sleep lab or hotel.


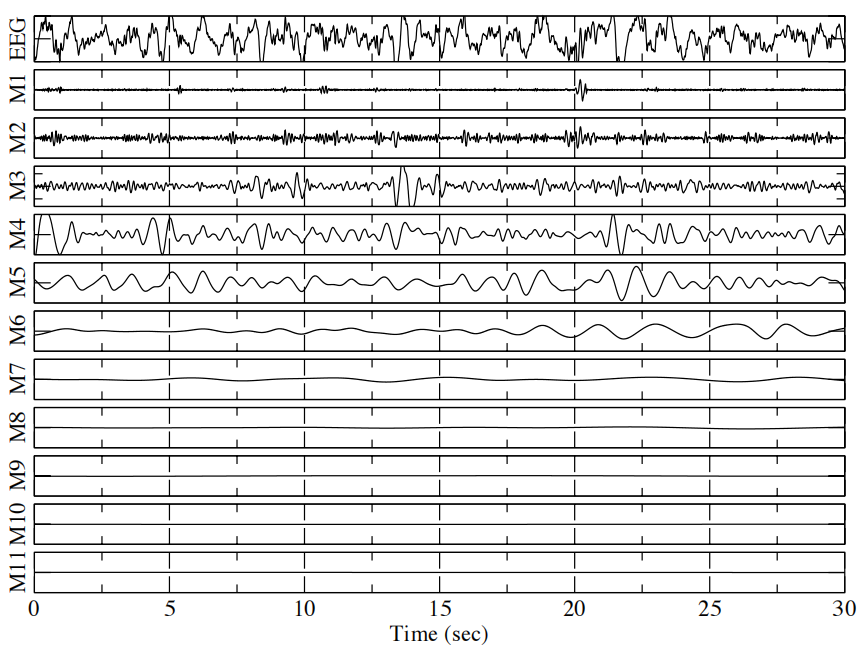


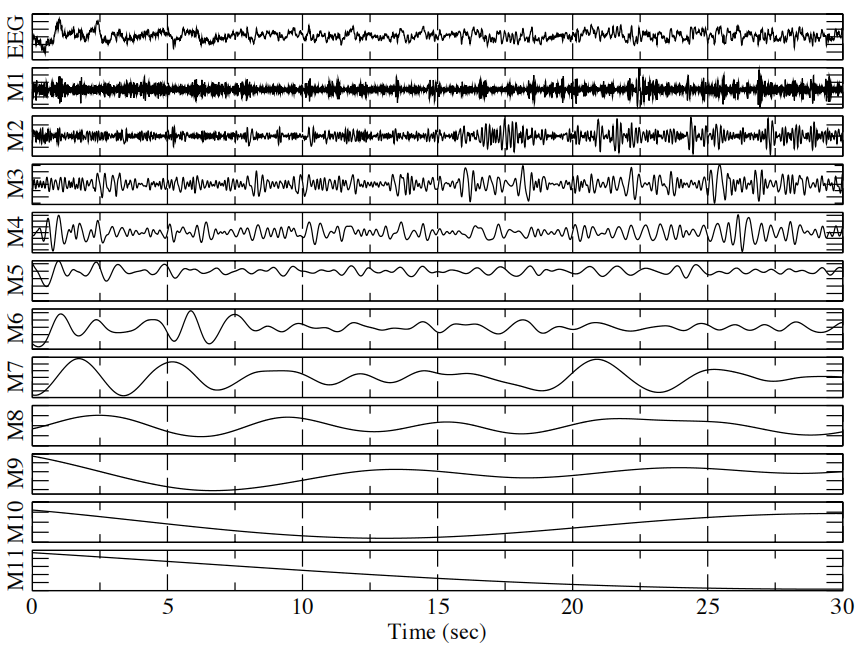


**Figure S5** EEG slow wave activity (SWA) and other oscillations can be separated as independent time series by empirical mode decomposition (EMD). Thirty seconds of single channel EEG signal during slow wave sleep (SWS, left) is shown in the top left panel. The bottom 11 panels show the 11 intrinsic mode functions (IMF1 to IMF11) decomposed by EMD. The frequency range for IMF3 to IMF5 is within the slow wave activity frequency, thus a large amplitude of these 3 IMFs indicates the presence of SWS. The frequency range for IMF1 and IMF 8, 9, 10 is 20-60Hz, 0.01-0.1 Hz respectively, all exhibit very low amplitude activity during SWS, and vice versa. The graph on the right shows thirty seconds of single channel EEG during N1 sleep. IMF3-5 has relatively smaller oscillations compared to IMF1 and IMF8-10, which indicates unstable sleep, i.e., non-SWS state.


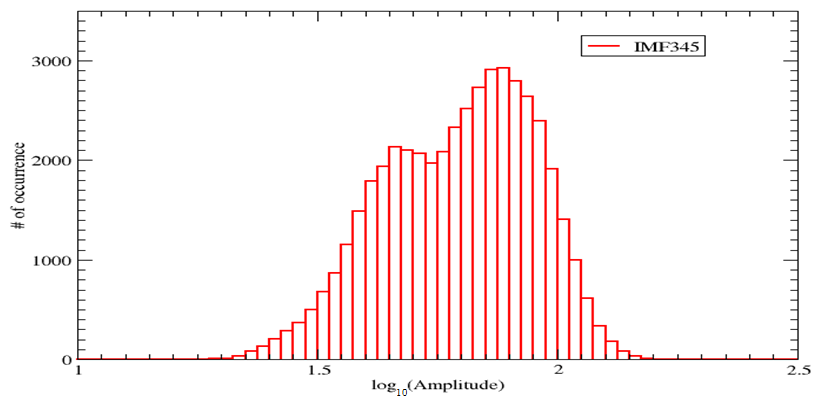


**Figure S6** Histogram of IMF3, 4, 5 amplitudes for an entire day and night sleep EEG recording of a healthy rat. Slow wave activities (SWA) of sleep embed a bimodal distribution. The average amplitude of IMF3, 4, 5, i.e., the main component of SWA, for every 1-second segment is calculated. The histogram is plotted on a semi-log graph, and exhibits bimodal distribution.

***Bimodal distribution of slow wave activity***

In this work, we proposed a cluster synchronization model to describe the emergence of SWS. A direct implication of the model is that, at any given instant, the system is either in the process of forming a dominant synchronized cluster or not, which should show up as a bimodal distribution if we sample the cluster size (which can be indirectly measured by the SWA amplitude) over time. Therefore, we examined the histograms of all subjects and, as expected, bimodal distribution can be visually observed in many subjects. However, we also noted that not all histograms show obvious bimodal pattern. This could be due to several factors of our proposed stochastic model, as we will discuss below.

**
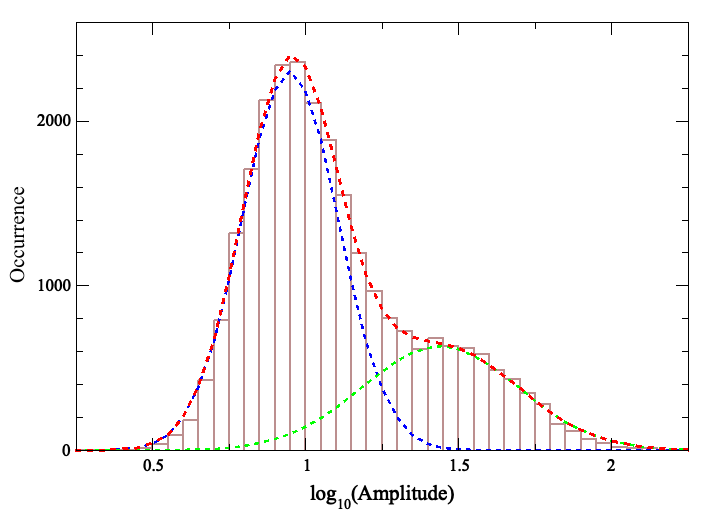
Figure S7** Histogram of IMF3,4,5 amplitude for an entire night sleep of a healthy 20-year-old female subject. The average amplitude of IMF3,4,5, i.e., the main component of SWA, for every 2-second segments is calculated. The histogram is plotted on a semi-log graph, and exhibits bimodal distribution that can be nicely fitted by the superposition (dashed red curve) of two log-normal distributions (dashed blue curve and dashed green curve). Since this figure is generated based on the entire night data, including REM sleep and wake duration, the non-SWS peak became much higher than the SWS peak.

For cases with smaller percentage of SWS, the second bump on the right side will be less prominent (Fig. S7). Furthermore, if we analyzed the data from the entire night (without removing REM sleep), the percentage of SWS will further decrease. Figure S7 shows the histogram of a different subject’s entire night data as an example where bimodal feature is not as clear. As we can see, the log-normal distribution that corresponds to the SWS state is much smaller than that of the non-SWS state, because this subject has less percentage of SWS, and also REM sleep is included in the analysis (to show the effect of including REM sleep in our analysis). Nevertheless, the histogram can still be very well fitted by the sum of two log-normal distributions (KS test with D < 0.03) suggested by our simple model.

As stated in the Discussion section, not all histograms from our database show clear evidence of bimodal distribution. However, we believe that the bimodal distribution is “hidden”, due to several possible stochastic factors as outlined in the Discussion section. For example, Fig. S8 is a case where there is no clear evidence of bimodal distributions. However, when our classification algorithm was applied, the entire NREM period was divided into SWS and non-SWS states, and we plotted the two corresponding histograms. Although there is significant overlap between these two histograms, by measuring the vagal tone index we confirmed that they are likely to be two different sleep states. Thus our algorithm can uncover the two underlying “hidden” distributions.

**Figure S8** Histogram of SWA amplitude (red vertical bar) for an entire night NREM sleep of a healthy young subject. By applying our algorithm, the NREM sleep can be divided into two classes (SWS vs. non-SWS). The histograms for SWS sleep (solid green curve) and non-SWS sleep (solid blue curve) show significant overlap.


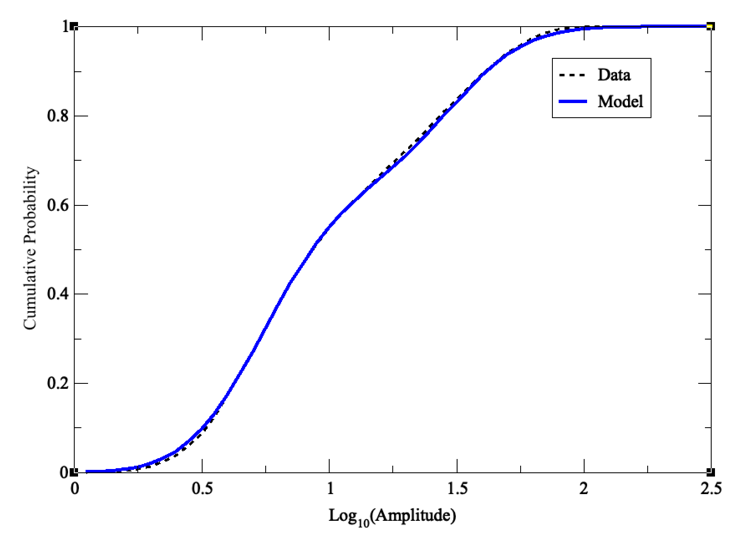
**Figure S9** We tested the goodness-of-fit of the theoretical model (superposition of two log-normal distributions, Fig 2 of the main text) with the Kolmogorov-Smirnov test (KS test). The cumulative probabilities of the data and the model are plotted in the left graph. As we can see, the deviations between these two curves are small. The maximal vertical distance between these two curves is 0.009. The P value of the KS test is 0.711 indicating that the data is not inconsistent with the two log-normal distributions. The Bayes factor is 0.153, indicating that the observed data are 6.5 times more likely under the null hypothesis than under the alternative hypothesis.

**Supplementary Discussion**

***Why EMD is applied for EEG analysis?***

Although, EMD is widely used in science and engineering research (according to Google Scholar, there are more than 23,000 citations of the original EMD article Ref. 19), it is less known in medical research where Fourier technique is the most popular time series analysis tool. Therefore, we briefly discuss the disadvantages of using Fourier based methods for EEG signal analysis, and the need to apply an adaptive technique such as the EMD method.

Fourier based analysis decomposes any signal into a collection of sinusoidal oscillations. For a simple oscillatory signal, for example, a triangle wave, that cannot be fitted by a single sinusoidal oscillation, then a series of “harmonic” sinusoidal waves will be introduced. Those harmonics (whose frequencies are integer multiples of the original triangle wave) are mathematically needed, but have no physical meaning. To address this general challenge of Fourier analysis, Hilbert transform was introduced and it can describe a sinusoidal oscillation with the instantaneous amplitude and frequency of the oscillation vary in time, i.e.,

$a\left( t \right)\sin(2\pi f\left( t \right)t)$ Eq. 1

However, Hilbert transform can only handle narrowband signal, specifically, it requires a well-defined frequency at any instant. When a signal is a superposition of multiple oscillations, and each has its own time-varying amplitude and frequency, i.e.,

$\sum a_{i}\left( t \right)\sin(2\pi f_{i}\left( t \right)t)$ Eq. 2

Direct application of Hilbert transform will lead to erroneous results. EMD was developed to solve this technical challenge (Ref. 19, 49). Briefly, EMD can decompose a complex signal into multiple components, called intrinsic mode functions (IMFs), and each IMF is an oscillatory function with amplitude and frequency that change with time, as defined in Eq. 1. This is completely different from the Fourier bandpass filter which will generate sinusoidal oscillations with constant amplitudes and frequencies (independent of time). We use the example below to illustrate this fundamental difference:

Figure S9 shows a simulated signal with asymmetric oscillation. The oscillation in the first half cycle (from peak to trough) is faster than the second half cycle (from trough to peak). We also added uncorrelated white noise to make it a more realistic broadband signal. Each complete cycle is 0.4 second (= 2.5 Hz frequency), which is in the slow wave frequency range. The simulated signal was generated with 120 Hz sampling frequency.


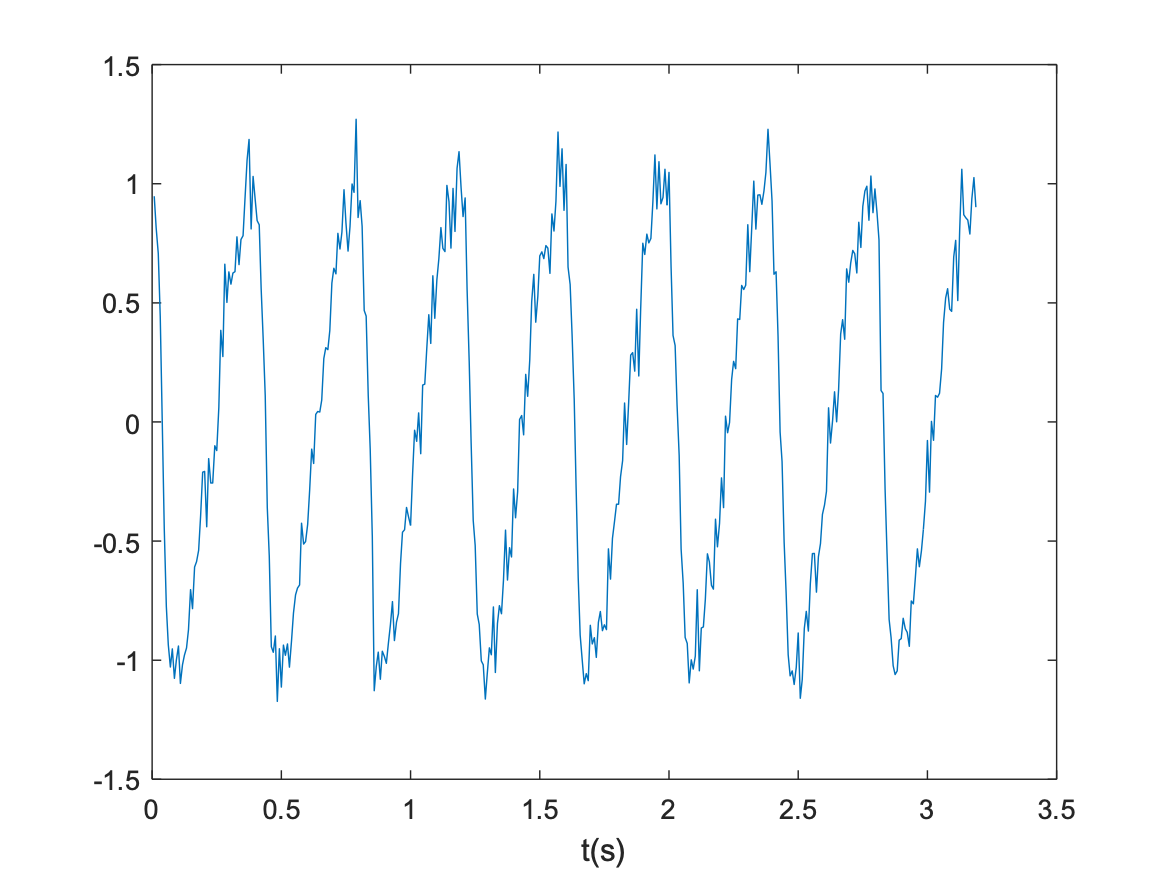


**Figure S10** A simulated signal with asymmetric oscillation.

With Fourier analysis, this non-sinusoidal signal is being decomposed into a set of sinusoidal oscillations, including one at 2.5 Hz (the fundamental frequency) and other harmonics (at 5Hz, 7.5Hz, 10Hz, etc.), Fig. S10 shows Fourier bandpass results, the fundamental oscillation with 2.5 Hz appears in the 1-4 Hz frequency band, however, this sinusoidal wave cannot fully account for the non-sinusoidal signal. As a result, about 10% of the original signal’s energy leaks into other frequencies (> 4 Hz). In comparison, the EMD analysis (see Fig. S11) can accurately keep the main oscillatory signal in one of the IMFs (IMF4 in this case), the energy leakage is less than 1%. Furthermore, almost all added noise has been decomposed into IMF1, with minimal effect on IMF4.


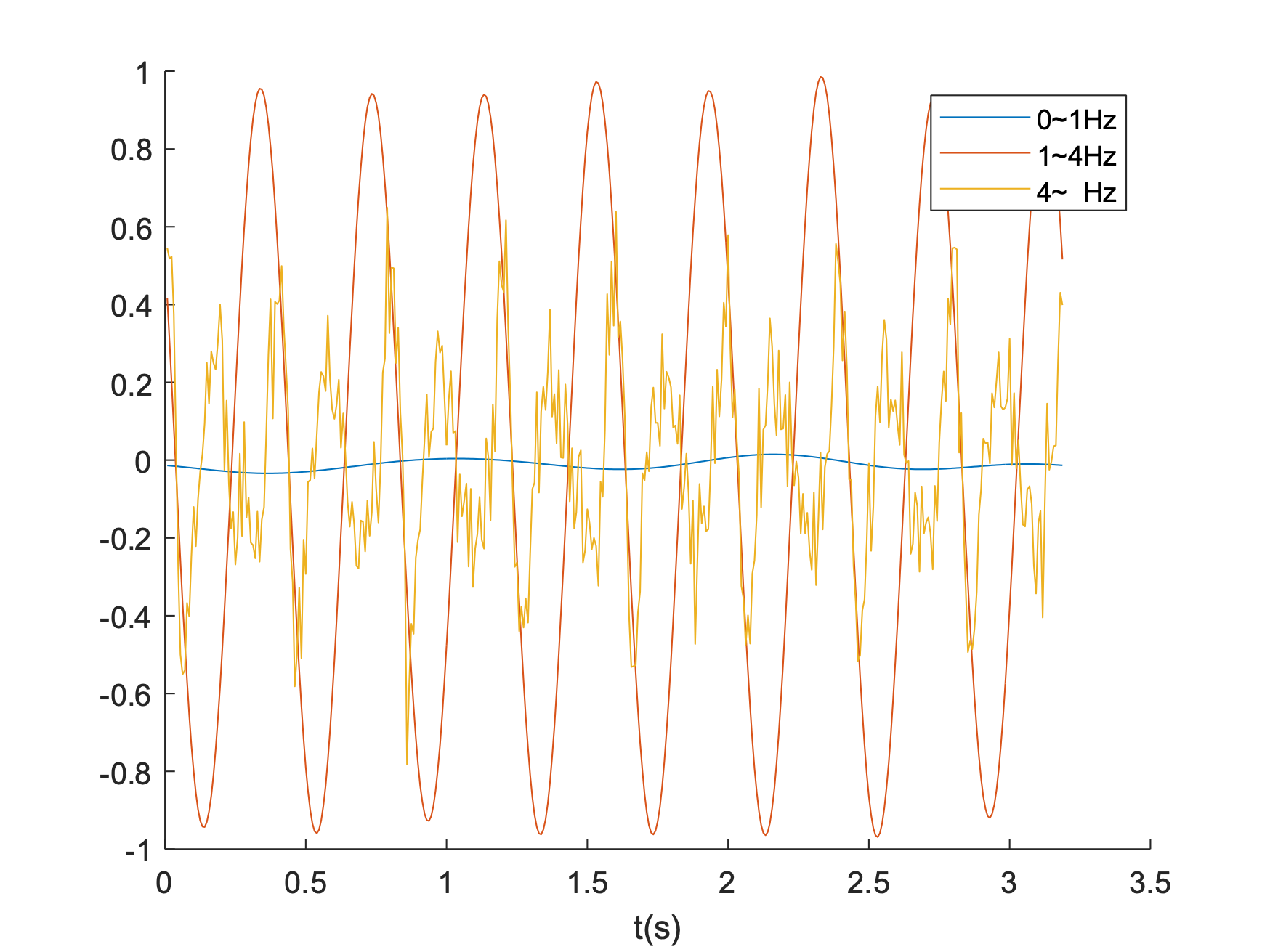


**Figure S11** Fourier analysis of the signal in Fig. S9.


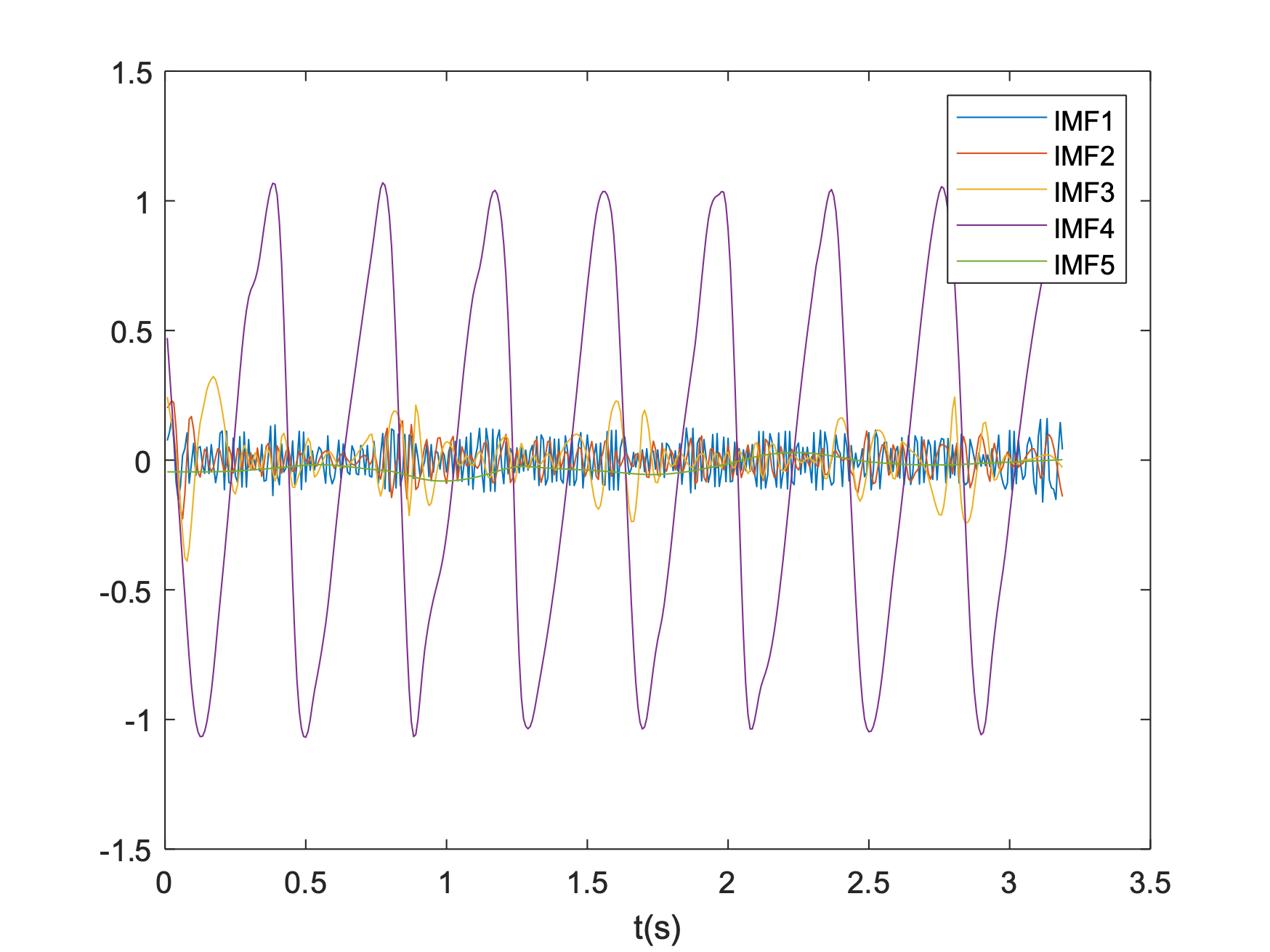


**Figure S12** EMD analysis of the signal in Fig. S9.

This simple example, an oscillatory signal with time-varying frequency within one cycle, shows the intrinsic limitation of Fourier analysis on non-stationary signal. If the amplitude is also nonstationary, as in almost all of the real-world complex signals, then the energy leakage will be even worse. Therefore, one cannot measure the slow wave amplitude accurately with any Fourier-based algorithms, since the basic assumptions of Fourier analysis require the signal to be stationary and linear. For our study, it is critical to measure the slow wave amplitude accurately, since all our results rely on its measurement.

It is known in the EMD literature that for a broadband signal, EMD behaves like a dyadic filter (but not Fourier based) with each level of decomposition. But the frequency is more flexible within each IMF, since it can be changed in time. Based on that general guideline, we estimated that the slow wave will most likely to be decomposed into IMF3 to IMF5. We have also verified this assumption by examining the EMD results for all data sets.

***Technical issues of the EMD-based SWS classification algorithm***

In this work, we analyzed the habitual sleep patterns of healthy subjects, so it is reasonable to assume that there are SWS periods in every data set. Furthermore, our algorithm also requires that large SWA amplitudes have to combine with small infra-slow oscillation amplitude in order for them to be classified. This combinational effect might be the reason that our algorithm is robust. However, for an extreme case when there is no SWS across the entire sleep period, then our algorithm will still falsely identify some segments as SWS. Therefore, we will need to refine our algorithm to take care of this issue. We believe the problem can be solved by introducing some additional post-processing steps, since those false events will appear randomly in time, and most of them will be isolated events of only 0.5 to 1 minute, thus we can filter them out easily. Future study is needed to systematically investigate how the accuracy of our algorithm will be affected when the percentage of true SWS approaches zero. For the current study, this is not an issue as for all the healthy subjects we analyzed, the lowest percentage of SWS is 18.3%. Furthermore, those classified SWS events do not show any evidence that they are randomly selected, as they exhibit the following 3 properties:

1. As we have shown in Fig. 4, these segments include almost all N3 stages, and some appear in N2 stage (called N2b periods) with many of them immediately before the beginning of N3 stages; but almost never appear in N1 stage.
2. The SWS events come in sequence, forming long consecutive segments of SWS, much longer than the expected segment length of a random process.
3. Perhaps most importantly for the thesis of this manuscript, we confirmed that SWS and non-SWS events classified by our algorithm exhibit different physiological properties. For example, as shown in Fig. S1, the vagal tone index is higher in the SWS periods compare to the non-SWS periods. This again suggests that the SWS events are not deduced by chance.

When there is no SWS in the entire data, there will still be oscillations in IMFs 3-5 due to background noise, since EEG are noisy signals to begin with. Similarly, when we apply Fourier analysis to this type of data, or other signals such as white noise, we will also find oscillations across all frequencies including slow wave frequency band (although no meaningful oscillation exist). However, the amplitude of the spurious slow wave oscillations obtained by the EMD will be much smaller than the amplitude derived from Fourier analysis since Fourier analysis distributes the energy of white noise uniformly over all frequencies, while the EMD analysis put most of the energy in IMF 1 (which has the highest frequency). Related investigations have been reported previously.^20, 22^
